# Serum uric acid and prostate cancer: findings from the NHANES (2007–2020)

**DOI:** 10.3389/fonc.2024.1354235

**Published:** 2024-10-25

**Authors:** Yu Yan, Hong Lin, Zhiyao He, Ling Wang

**Affiliations:** ^1^ Department of Pharmacy, West China Hospital, Sichuan University, Chengdu, China; ^2^ Key Laboratory of Drug-Targeting and Drug Delivery System of the Education Ministry, Department of Clinical Pharmacy and Pharmacy Administration, West China School of Pharmacy, Sichuan University, Chengdu, China

**Keywords:** prostate cancer, serum uric acid, curve fitting, cross-sectional study, logistic regression, NHANES

## Abstract

**Background:**

The relationship between serum uric acid (SUA) levels and prostate cancer (PCa) remains controversial. This cross-sectional study investigated the association between SUA levels and PCa incidence.

**Methods:**

A total of 9,776 participants aged ≥40 from the National Health and Nutrition Examination Survey (NHANES) from 2007 to 2020 were included, 503 of whom had PCa. Weighted univariate logistic regression, multivariate logistic regression, and smooth-fitting curve analyses were used to analyze the association between SUA and PCa incidence. Concurrently, the fitted smoothing curves were used to explore the potential non-linear relationships. If non-linearity was observed, a recursive algorithm further calculated the inflection point.

**Results:**

Three models were used to analyze the correlation between SUA levels and PCa incidence. All regression models demonstrated a negative correlation between SUA levels and PCa incidence (model 1: OR = 0.88, 95% CI=0.80–0.97; model 2: OR = 0.87, 95% CI=0.80–0.96; model 3: OR = 0.87, 95% CI=0.78–0.96). According to the trend test, with increasing SUA, the risk of PCa showed a downward trend (three models P for trend = 0.037, 0.015, 0.016). According to the subgroup analysis, a significant negative correlation between SUA and PCa was detected in individuals aged >60 years, non-Hispanic whites, those of other races, and those with hypertension. Moreover, the association between SUA and PCa followed a U-shaped curve among participants without hypertension, and the inflection point of SUA was 5.1 mg/dl.

**Conclusions:**

This cross-sectional study revealed a negative relationship between SUA levels and the risk of PCa, particularly in specific demographic groups. These findings offer a fresh perspective on the role of SUA in PCa patients, potentially paving the way for new approaches for the prevention and treatment of PCa. However, further studies are necessary to validate these findings.

## 1 Introduction

Prostate cancer (PCa) is one of the most common cancers in men and the fifth leading cause of cancer death globally, with an estimated 1,414,000 new cancer cases and 375,304 deaths in 2020. PCa is the most frequently diagnosed cancer in 112 countries and the leading cause of cancer death in 48 countries (1, 2). Therefore, the disease burden of PCa is expected to increase steadily due to the aging population and economic growth (3). To date, little is known about the causes of PCa. The established risk factors for PCa include age, family history, genetic mutations, and certain diseases (4). Thus, it is necessary to explore whether there are potentially unknown risk factors for PCa to prevent and control its occurrence and development.

Serum uric acid (SUA) is a product of purine metabolism degradation (5). The oxidation of xanthine and hypoxanthine produces SUA by xanthine oxidoreductase (XOR) (6, 7). Earlier studies have linked high SUA levels to several cardiovascular diseases and metabolic syndromes (8). Some recent findings suggest a correlation between SUA and cancer (9). High blood concentrations of SUA can lead to gout. Gout is a form of arthritis arising from SUA accumulation in the bloodstream (10) and is associated with an elevated risk for cancer overall. Gout-associated chronic inflammation, in turn, is associated with increased cancer risk. Another possible explanatory mechanism for the association between gout and carcinogenesis could be genetic instability induced by oxidative stress (11). However, in specific cancer types, the impact of SUA on PCa is still controversial (12, 13). One study showed low SUA levels and increased inflammatory markers; therefore, low SUA levels were risk factors for PCa (14). In contrast, the results of another study suggested that higher SUA levels increase PCa risk (4). Accordingly, we aimed to investigate the association between SUA levels and PCa incidence based on data from the US National Health and Nutrition Examination Survey (NHANES).

## 2 Methods

### 2.1 Data sources and study population

A cross-sectional study method was applied in this study. The study data were collected from the 2007–2020 NHANES datasets (including data from seven 2-year circles). The NHANES is designed to assess health and nutritional status in the U.S. The ethics protocol for the NHANES was approved by The National Center for Health Statistics Research Ethics Review Board, and informed consent was obtained from all participants (15, 16). Weighted statistics were used in this study following National Center for Health Statistics (NCHS) guidelines, and the survey weights were recommended by the NHANES, which combines factors such as sampling probability, non-response adjustment, and *post-hoc* stratification to ensure the representativeness of the data (relevant instructions and formulas have been added to the manuscript, see https://wwwn.cdc.gov/nchs/nhanes/tutorials/weighting.aspx; the formula used is Weight=if sddsrvyr in (5,6,7,8,9,10) then MEC12YR = 1/6 * WTMEC2YR), where the weight analysis method helps to reflect the actual situation of the U.S. population more accurately.

**Figure 1:**
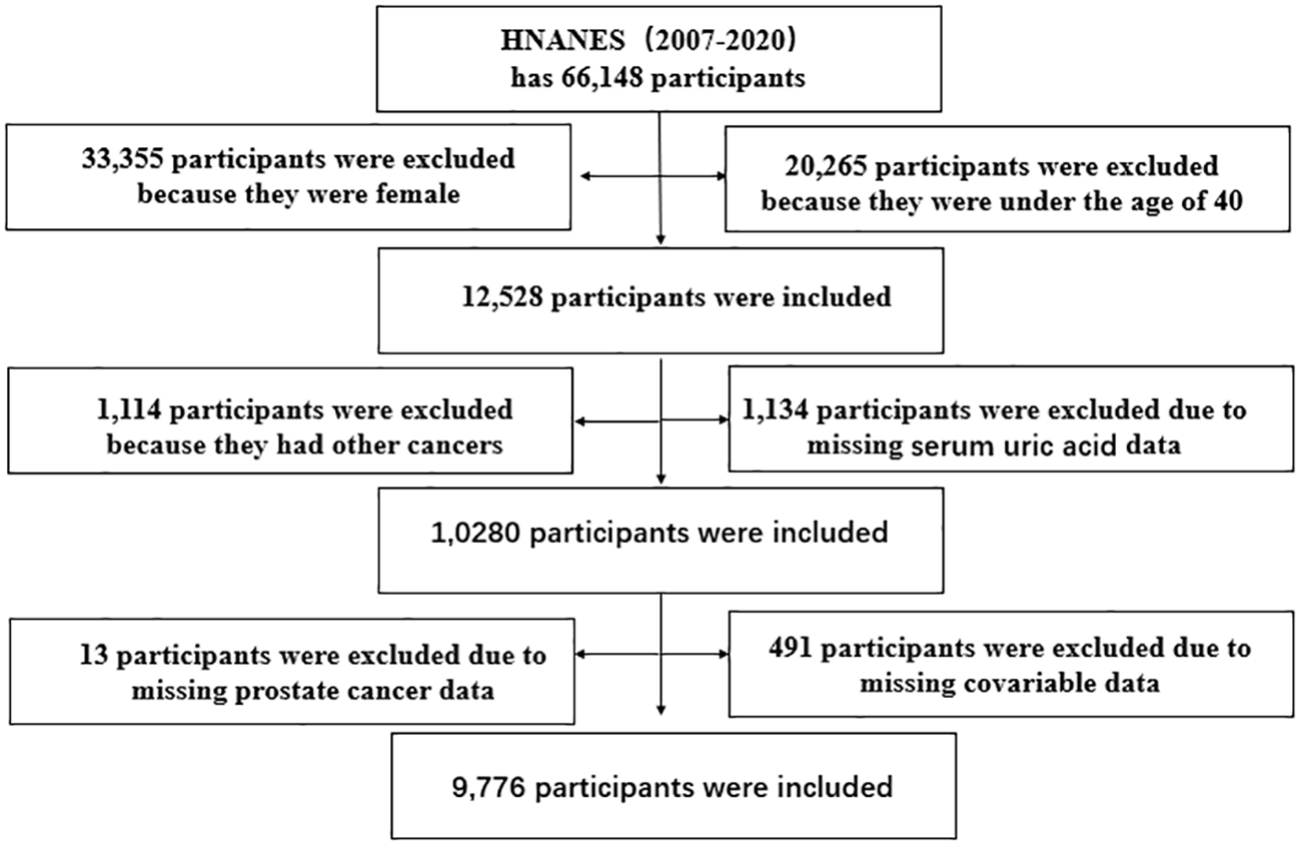
Flowchart of participant selection.

In our study, 66,148 participants were initially selected, and 9,776 men were eventually enrolled in the data analysis (503 patients with PCa); female participants, participants under 40 years old, and those suffering from other cancers were excluded, and those lacking PCa, SUA, and waist circumference (WC) data were excluded. The specific inclusion and exclusion criteria are shown in **Figure 1**.

### 2.2 Study variables

All the study variables were collected from well-trained health professionals. All study methods can be downloaded from the NHANES website. The experimental data and laboratory variables were measured following the technical standards issued by the NHANES. The laboratory program manual provided by the NHANES described the exposure variables SUA, mg/dl, and SUA detection methodology in detail.

#### 2.2.1 Outcome variable

PCa incidence was defined according to the participants’ self-reports. When doctors or other health professionals were asked whether they had PCa or prostate malignancy, they responded “Yes”. Complete informed consent was obtained from all participants before the interview and inspection took place.

#### 2.2.2 Covariates

Covariates were determined based on previous studies and our clinical experience and included population statistical data (such as age, race, education, and poverty–income ratio) (16–19) and human measurements (such as body mass index (BMI), WC, and blood pressure). Health-related behaviors (smoking, drinking, physical activity, diabetes mellitus (DM), hypertension, stroke, and coronary heart disease (CHD)). Biochemistry (albumin, ALT, AST, BUN, SCR, total bilirubin (Tbil), total protein, TG, globulin, and total cholesterol (TC)). The diagnostic criteria for diabetes mellitus included the following: (1) self-reported diabetes mellitus, (2) glycosylated hemoglobin ≥6.5%, (3) fasting blood glucose (F.D.G. ≥126 mg/dl), and (4) taking insulin or oral hypoglycemic drugs. The diagnostic criteria for hypertension included the following: (1) systolic blood pressure ≥130 mmHg or diastolic pressure ≥80 mmHg, (2) self-reported hypertension, and (3) blood pressure medication ^use^ (20, 21).

### 2.3 Statistical analyses

The statistical software R (version 4.2.0) and EmpowerStats were used to analyze all the study data. Continuous variables are presented as the means ± SDs (normally distributed data are represented by the median and interquartile range (IQR)). P values were calculated using a weighted linear regression model. The differences between groups for categorical variables were calculated by the chi-square test, with P<0.05. The Epidemiology Strobe guide used weighted single-variable and multivariable logistic regression to analyze the relationship between SUA levels and PCA (22).

Three regression models based on the different confounding factors were created in this study: model 1 (unadjusted model); model 2 (adjusted for age, race, education, poverty, income ratio, BMI adjustment); and model 3 (modified for age, race, education, PIR, BMI, smoking status, drinking status, physical activity, WC, albumin, ALT, AST, BUN, S.C.R., total protein, TG, total protein, globulin, HBP, DM, stroke, CHD).

Furthermore, smooth curve fitting and generalized additive models revealed a non-linear relationship between SUA levels and PCa risk. When non-linearity was detected, a recursive algorithm was applied to calculate the inflection point in the correlation between SUA levels and PCa risk (23).

## 3 Results

### 3.1 Characteristics of the population

Our study included 9,776 participants; the average age was 58.9 ± 11.8 years. We divided all the participants into a healthy group (9,273 cases) and a PCa group (503 cases). **Table 1** shows the basic characteristics of all participants included in this study and **Table 2** shows the Healthcare diagnosis data for study participants for all participants. Then, we compared the differences in each variable between the two groups. The results of this study showed that age, race, education, poverty income ratio, smoking status, drinking status, physical activity, albumin, ALT, AST, BUN, SCR, total protein, globulin, SUA, HBP, DM, stroke, and stroke were significantly different between the two groups. WC, TBil, globulin, and BMI were not significantly different between the two groups (**Tables 1**, **2**).

**Table 1:** Population characteristics data of study participants.

Variable	Total	Is there PCa?		P-value
		No	Yes	
Cases	9,776	9,273	503	
Age, mean ± SD (years)	58.9 ± 11.8	58.1 ± 11.6	71.9 ± 7.6	<0.001
Race, n (%)				<0.001
Mexican American	1,398 (14.3)	1,374 (14.8)	24 (4.8)	
Non-Hispanic White	3,946 (40.4)	3,678 (39.7)	268 (53.3)	
Non-Hispanic Black	2,233 (22.8)	2,081 (22.4)	152 (30.2)	
Other Race	2,199 (22.5)	2,140 (23.1)	59 (11.7)	
Education, n (%)				0.003
Lower than high school	2,677 (27.4)	2,576 (27.8)	101 (20.1)	
High school diploma	2,278 (23.3)	2,166 (23.4)	112 (22.3)	
Higher than high school	4,821 (49.3)	4,531 (48.9)	290 (57.7)	
Poverty income ratio, n (%)				0.009
<1.3	2,492 (25.5)	2,410 (26.0)	82 (16.3)	
1.3–3.5	3,283 (33.6)	3,071 (33.1)	212 (42.1)	
>3.5	3,038 (31.1)	2,875 (31.0)	163 (32.4)	
Missing data	963 (9.9)	917 (9.9)	46 (9.1)	
Smoking status, n (%)				<0.001
Never	4,215 (43.1)	4,010 (43.2)	205 (40.8)	
Quitting	3,452 (35.3)	3,211 (34.6)	241 (47.9)	
Current	2,109 (21.6)	2,052 (22.1)	57 (11.3)	
Drinking status, n (%)				<0.001
Never	539 (5.5)	505 (5.4)	34 (6.8)	
Moderate	3,384 (34.6)	3,155 (34.0)	229 (45.5)	
Heavy	3,497 (35.8)	3,380 (36.4)	117 (23.3)	
Missing data	2,356 (24.1)	2,233 (24.1)	123 (24.5)	
Physical activity, n (%)				<0.001
Vigorous	3,133 (32.0)	2,967 (32.0)	166 (33.0)	
Moderate	2,878 (29.4)	2,680 (28.9)	198 (39.4)	
Light	3,765 (38.5)	3,626 (39.1)	139 (27.6)	

**Table 2:** Healthcare diagnosis data for study participants.

Variable*	Total	Is there PCa?		P-value
		No	Yes	
Cases	9,776	9,273	503	
WC, mean ± SD (cm)	103.7 ± 14.9	103.6 ± 14.9	105.4 ± 14.2	0.079
Albumin, mean ± SD (g/dL)	4.2 ± 0.3	4.2 ± 0.3	4.2 ± 0.3	<0.001
ALT, median (IQR) (U/L)	23.0 (18.0-31.0)	23.0 (18.0-31.0)	20.0 (16.0-26.0)	<0.001
AST, median (IQR) (U/L)	24.0 (20.0-29.0)	24.0 (20.0-29.0)	23.0 (20.0-27.0)	0.002
BUN, mean ± SD (mg/dL)	15.4 ± 6.4	15.3 ± 6.3	17.8 ± 7.7	<0.001
SCR, median (IQR) (mg/dL)	1.0 (0.8-1.1)	(0.8-1.1)	1.1 (0.9-1.2)	<0.001
TBil, mean ± SD (µmol/L)	0.7 ± 0.3	0.7 ± 0.3	0.7 ± 0.3	0.501
Total protein, mean ± SD (g/dL)	7.2 ± 0.5	7.2 ± 0.5	7.1 ± 0.5	<0.001
TG, median (IQR) (mg/dL)	134.0 (89.0-209.0)	134.0 (90.0-210.0)	124.0 (84.0-183.0)	0.011
Globulin, mean ± SD (g/dL)	2.9 ± 0.5	2.9 ± 0.5	2.9 ± 0.5	0.075
TC, mean ± SD (mg/dL)	191.0 ± 43.1	191.5 ± 43.1	180.7 ± 41.5	<0.001
SUA, mean ± SD (mg/dL)	6.0 ± 1.4	6.0 ± 1.4	6.0 ± 1.4	0.011
BMI, n (%)				0.338
<25.0	2,238 (22.9)	2,111 (22.8)	127 (25.2)	
25.0–29.9	3,946 (40.4)	3,736 (40.3)	210 (41.7)	
>29.9	3,592 (36.7)	3,426 (36.9)	166 (33.0)	
HBP, n (%)				<0.001
No	3,203 (32.8)	3,101 (33.4)	102 (20.3)	
Yes	6,573 (67.2)	6,172 (66.6)	401 (79.7)	
DM, n (%)				<0.001
No	7,058 (72.2)	6,724 (72.5)	334 (66.4)	
Yes	2,718 (27.8)	2,549 (27.5)	169 (33.6)	
Stroke, n (%)				<0.001
No	9,281 (94.9)	8,836 (95.3)	445 (88.5)	
Yes	495 (5.1)	437 (4.7)	58 (11.5)	
Stroke, n (%)				<0.001
No	9,035 (92.4)	8,598 (92.7)	437 (86.9)	
Yes	741 (7.6)	675 (7.3)	66 (13.1)	

*WC, waist circumference; ALT, alanine transaminase; AST, aminotransferase; BUN, blood urea nitrogen; SCR, serum creatinine; Tbil, total bilirubin; TC, total cholesterol; TG, triglyceride; BMI, body mass index, HBP, high blood pressure; DM, diabetes mellitus; CHD, coronary heart disease; PCa, prostate cancer.

### 3.2 Univariate analysis of factors related to PCa

The weighted univariate analysis revealed that age, WC, BUN, SCR, HBP, DM, stroke status, and CHD status were positively associated with PCa incidence (risk factors). Albumin, ALT, and total protein were negatively correlated with PCa incidence and were protective factors. WC, BMI, AST, total bilirubin (Tbil globulin), and total cholesterol (TC) were not significantly different. However, there were different conclusions among the subgroups (**Table 3**).

**Table 3:** Univariate analysis of factors associated with PCa, weighted.

Variable*	Prostate cancer	Variable*	Prostate cancer
	OR (95% CI)		OR (95% CI)
Age	1.14 (1.13-1.16)	WC	1.01 (1.00-1.02)
RACE		B.M.I.	
Mexican American	Ref.	<25.0	Ref.
Non-Hispanic White	3.75 (2.64-5.31)	25.0–29.9	0.92 (0.69-1.21)
Non-Hispanic Black	4.76 (3.24-6.98)	>29.9	0.77 (0.55-1.07)
Other Race	2.13 (1.19-3.82)	Albumin	0.42 (0.29-0.61)
Education		ALT	0.97 (0.95-0.98)
Lower than high school	Ref.	AST	0.99 (0.97-1.00)
High school diploma	1.13 (0.78-1.62)	BUN	1.06 (1.04-1.07)
Higher than high school	1.56 (1.16-2.11)	SCR	1.31 (1.16-1.49)
Poverty income ratio		TBil	0.87 (0.57-1.33)
<1.3	Ref.	Total protein	0.51 (0.39-0.69)
1.3–3.5	2.05 (1.35-3.11)	TG	1.00 (1.00-1.00)
>3.5	1.55 (1.07-2.25)	Globulin	0.79 (0.61-1.03)
Missing data	1.76 (1.08-2.87)	TC	0.99 (0.99-1.00)
Smoking status		HBP	
Never	Ref.	NO	Ref.
Quitting	1.64 (1.26-2.14)	YES	1.79 (1.29-2.49)
Current	0.54 (0.34-0.85)	DM	
Drinking status		NO	Ref.
Never	Ref.	YES	1.70 (1.33-2.18)
Moderate	1.29 (0.76-2.19)	Stroke	
Heavy	0.49 (0.27- 0.89)	NO	Ref.
Missing data	0.96 (0.57-1.63)	YES	3.18 (2.03-5.00)
Physical activity		CHD	
Vigorous	Ref.	NO	Ref.
Moderate	1.55 (1.17-2.06)	YES	2.13 (1.47-3.09)
Light	0.52 (0.38-0.72)		

*WC, waist circumference; ALT, alanine transaminase; AST, aminotransferase; BUN, blood urea nitrogen; SCR, serum creatinine; Tbil, total bilirubin; TC, total cholesterol; TG, triglyceride; BMI, body mass index; HBP, high blood pressure; DM, diabetes mellitus; CHD, coronary heart disease; PCa, prostate cancer.

### 3.3 Multivariate analysis of SUA and related factors in PCa patients

**Table 4** lists the results of the three regression models. In model 1, SUA was negatively correlated with PCa incidence (OR = 0.88, 95% CI=0.80–0.97), and for each 1-mg/dl increase in SUA, the risk of PCa decreased by 12%. In model 2, SUA was negatively correlated with PCa incidence (OR = 0.87, 95% CI=0.80–0.96), and the risk of PCa decreased by 13% for every 1-mg/dl increase in SUA. In model 2, SUA was inversely associated with PCa incidence (OR = 0.87, 95% CI=0.78–0.96), with each 1-mg/dl increase in SUA associated with a 13% decrease in the risk of PCa.

**Table 4:** Multiple logistic regression analysis of SUA and PCa, weighted.

Variable	Model 1*	Model 2**	Model 3***
	OR (95% CI)	OR (95% CI)	OR (95% CI)
SUA (mg/dl)	0.88(0.80, 0.97)	0.87(0.80, 0.96)	0.87 (0.78, 0.96)
SUA group			
Q1	Ref.	Ref.	Ref.
Q2	0.65(0.46, 0.91)	0.75(0.51, 1.08)	0.76 (0.51, 1.13)
Q3	0.63(0.42, 0.95)	0.69(0.45, 1.07)	0.68 (0.44, 1.05)
Q4	0.69(0.50, 0.95)	0.65(0.46, 0.92)	0.64 (0.45, 0.93)
P for trend	0.037	0.015	0.016

*Model 1: unadjusted.

**Model 2: adjust for age, race, education, PIR, and BMI.

***Model 3: adjust for age, race, education, PIR, BMI, smoking status, drinking status, physical activity, WC, albumin, ALT, AST, BUN, S.C.R., Tbil, total protein, TG, TC, globulin, HBP, DM, Stroke, CHD.

ALT, alanine transaminase; AST, aminotransferase; BUN, blood urea nitrogen; SCR, serum creatinine; Tbil, total bilirubin; TC, total cholesterol; TG, triglyceride; BMI, body mass index, HBP, high blood pressure; DM, diabetes mellitus; CHD, coronary heart disease.

**Figure 2:**
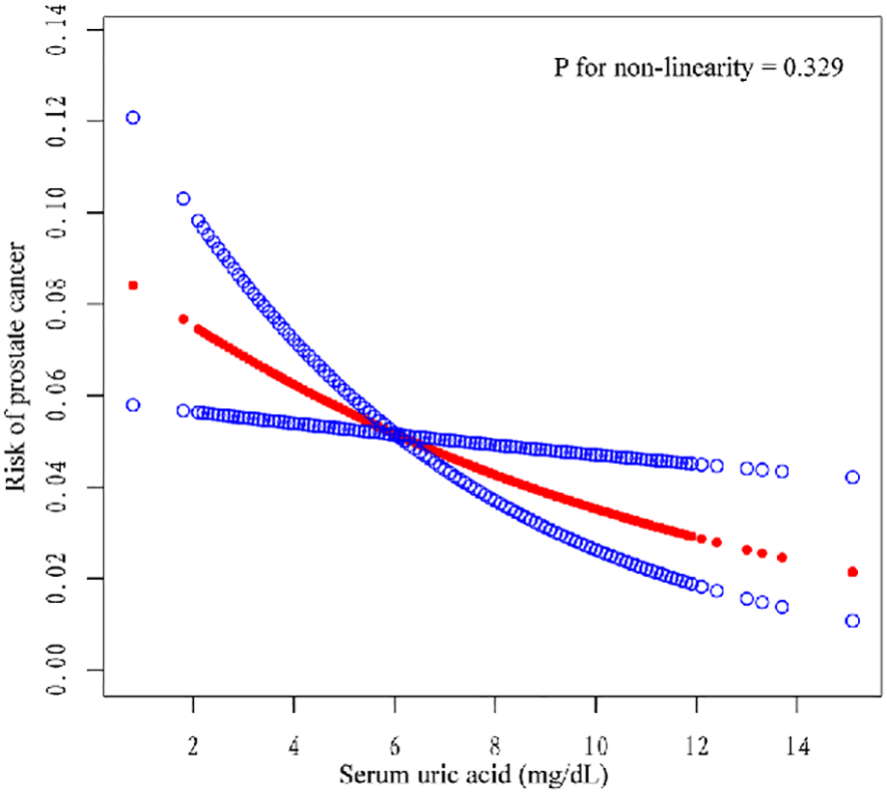
Curve fitting of SUA and PCa (P for non-linearity = 0.329).

We also conducted subgroup analysis for all the models. In unadjusted model 1, compared with Q1, Q2, Q3, and Q4 were negatively correlated (OR = 0.65, 95% CI=0.46–0.91; OR = 0.63, 95% CI=0.42–0.95; OR = 0.69, 95% CI=0.50–0.95). In model 2, Q4 was negatively correlated with Q1 (OR= 0.65, 95% CI=0.46–0.92). In model 3, Q4 was negatively correlated with Q1 (OR= 0.64, 95% CI=0.45–0.93). The risk of PCA decreased with increasing SUA in all models, and this trend was consistent (three models P for trend = 0.037, 0.015, 0.016). Moreover, we constructed a smooth curve to observe the non-linear relationship between SUA and PCa (P for non-linearity=0.329) (**Figure 2**).

### 3.4 Subgroup analysis of the correlation between SUA and PCa incidence status

We performed a stratified subgroup analysis of age, race, and HBP to further analyze whether the relationship between SUA and PCa existed stably in the different subgroups (**Table 5**). In the >60-year age group, there was a negative correlation (OR = 0.87, 95% CI=0.80–0.95; OR = 0.87, 95% CI=0.79–0.95; OR = 0.85, 95% CI=0.76–0.94) in all three models. In the race group, non-Hispanic White ethnicity and other races were negatively correlated according to the three models (OR = 0.85, 95% CI=0.75–0.96; OR = 0.87, 95% CI=0.78–0.98; OR = 0.85, 95% CI=0.75–0.97; and OR = 0.76, 95% CI=0.59, 0.99; OR = 0.71, 95% Cl=0.54, 0.93; OR = 0.69, 95% Cl=0.54, 0.90). In the HBP group, hypertension was negatively correlated with hypertension according to all the models (OR = 0.84, 95% CI=0.76–0.93; OR = 0.86, 95% CI=0.78–0.95; OR = 0.87, 95% CI=0.77–0.98) (**Table 5** for details).

**Table 5:** The relationship between SUA and PCa, stratified by age, race, and high blood pressure, was weighted.

Exposure variables	Model 1*	Model 2**	Model 3***
	OR(95% CI)	OR(95% CI)	OR(95% CI)
Subgroup analysis stratified by			
Age			
≤60	0.98(0.66, 1.44)	0.91(0.62, 1.33)	0.85(0.58, 1.25)
>60	0.87(0.80, 0.95)	0.87(0.79, 0.95)	0.85(0.76, 0.94)
Race			
Mexican American	0.88(0.61, 1.25)	0.99(0.72, 1.35)	1.19(0.81, 1.75)
Non-Hispanic White	0.85(0.75, 0.96)	0.87(0.78, 0.98)	0.85(0.75, 0.97)
Non-Hispanic Black	1.02(0.90, 1.15)	0.97(0.85, 1.11)	0.99(0.85, 1.16)
Other race	0.76(0.59, 0.99)	0.71(0.54, 0.93)	0.69(0.54, 0.90)
HBP			
NO	0.95(0.72, 1.26)	0.92(0.69, 1.23)	0.87(0.62, 1.23)
YES	0.84(0.76, 0.93)	0.86(0.78, 0.95)	0.87(0.77, 0.98)

*Model 1: unadjusted.

**Model 2: adjust for age, race, education, PIR, and BMI.

***Model 3: adjust for age, race, education, PIR, BMI, smoking status, drinking status, physical.

ALT, alanine transaminase; AST, aminotransferase; BUN, blood urea nitrogen; SCR, serum creatinine; Tbil, total bilirubin; TC, total cholesterol; TG, triglyceride; BMI, body mass index, HBP, high blood pressure; DM, diabetes mellitus; CHD, coronary heart disease.

**Figure 3:**
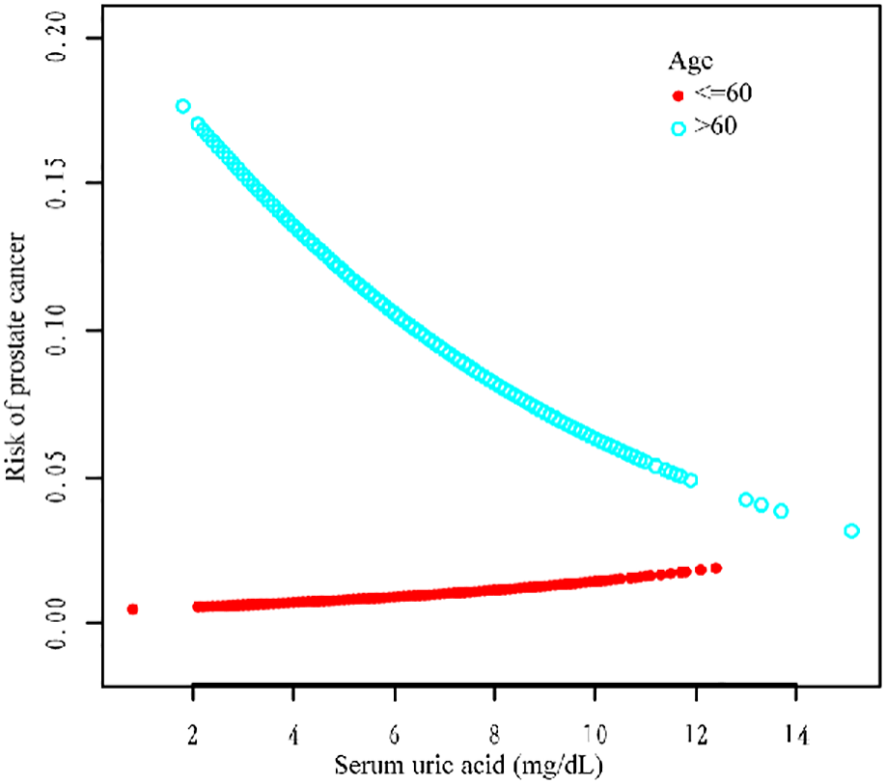
Association between SUA and PCa stratified by age.

**Figure 4:**
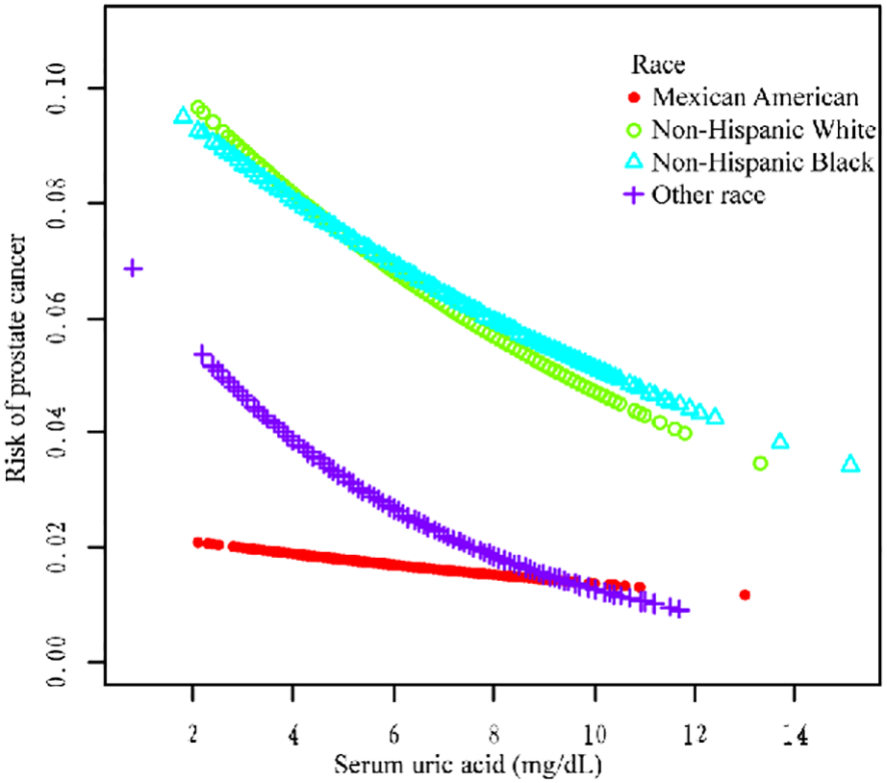
Association between SUA and PCa stratified by race.

**Figure 5:**
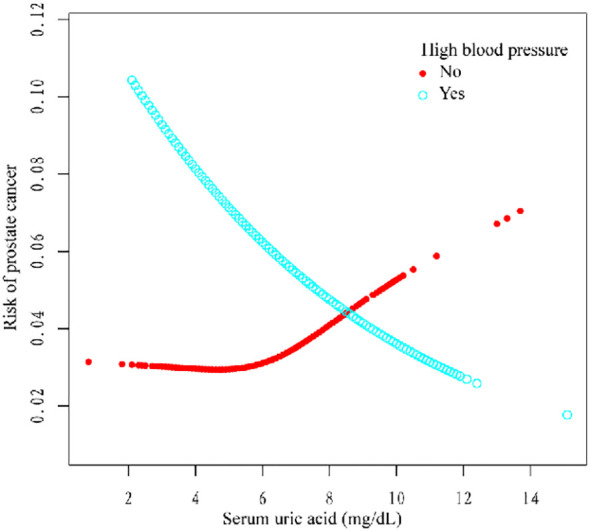
Association between SUA and PCa stratified by high blood pressure.

Additionally, we used a smoothing curve to explore the potential non-linear relationships among age, race, and HBP (**Figures 3**–**5**). The results showed that the association between SUA and PCa followed a U-shaped curve among participants without hypertension, and the inflection point of SUA was 5.1 mg/dl (**Table 6**).

**Table 6:** The results of a two-piecewise linear regression model between serum uric acid and prostate cancer.

Serum uric acid		Adjusted OR (95% CI)
No high blood pressure		
The inflection point of serum uric acid		5.1
Regression coefficients (≤inflection point)		0.73 (0.42, 1.27)
Regression coefficients (>inflection point)		1.13 (0.91, 1.41)
P for log-likelihood ratio tests		0.208

Adjust for age, race, education, PIR, BMI, smoking status, drinking status, physical activity, WC, albumin, ALT, AST, BUN, S.C.R., TBil, total protein, TG, TC, globulin, DM, Stroke, CHD.

## 4 Discussion

A negative relationship between SUA level and the risk of PCa was found in this cross-sectional study, particularly in the age >60 years, non-Hispanic white, other race, and hypertension subgroups. In addition, the correlation between SUA and PCa followed a U-shaped curve among the groups without hypertension, and the inflection point of SUA was 5.1 mg/dl. This study was the first to investigate the relationship between SUA and PCa based on data from the NHANES. SUA is a protective factor in PCa patients, and with increasing SUA levels, the risk of PCa decreases.

At present, the relationship between SUA and PCa is still controversial. Several studies have reported that SUA protects against cancer by increasing antioxidant capacity (9). Nevertheless, other study results contradict the proposed antioxidant and protective effects of SUA against cancer and suggest that high SUA concentrations are associated with outcomes, possibly reflecting more serious prognostic indications (24). Another population-based cohort study revealed a J-shaped relationship between the baseline UA level and PCa-related mortality risk (25). There are two possible explanations for these different conclusions. First, some preliminary research has missed potential variables that may result in different outcomes. However, this study included mostly common risk factors for PCa as covariates to increase the accuracy of the results. Second, some studies included only a few PCa patients, which may have led to the lack of relevance. This study uses a weighted analysis method to represent the data of American men aged more than 40 years from 2007 to 2020. Therefore, a larger sample size was included to support the conclusions of this study. Therefore, the conclusions of this study have additional credibility.

For further analysis, we also conducted a subgroup analysis. The results of the subgroup analysis showed that SUA and PCa had a significant negative association in the age >60, non-Hispanic white, other race, and hypertension subgroups. Moreover, the association between SUA and PCa followed a U-shaped curve among participants without hypertension, and the inflection point of SUA was 5.1 mg/dl. Several studies have shown a correlation between SUA and high blood pressure (26). Hyperuricemia (an SUA level greater than 6.8 mg/dl) was associated with an increased risk of uncontrolled hypertension and resistance to antihypertensive therapy (27–29). However, the relationship between hypertension and PCa still needs further exploration.

Something that needs special clarification: The SUA level is not as high as possible; hyperuricemia can also damage health. Therefore, this study’s conclusions may apply to protecting against PCa at a higher uric acid concentration in the normal range, especially among specific people, such as those aged >60 years, non-Hispanic whites, other races, and those with hypertension. However, additional studies need to be conducted to verify these conclusions.

### 4.1 Potential mechanism

SUA may act as an antioxidant by scavenging reactive oxygen species (ROS) (29). ROS scavenging can reduce the oxidative stress-induced apoptosis of cancer cells, thereby promoting their growth and survival (30). One study reported that SUA plays a major role in stimulating immune cells (31). This effect of SUA may be due to its protective effect against malignancy. In brief, a reduction in SUA levels may be related to cancer, on the one hand, through reduced antioxidant capacity and, on the other hand, through suppression of the immune system. These studies all support the conclusion that SUA is a protective factor against cancer, which is also consistent with the conclusion of this study.

### 4.2 Strength of this study

Compared with previous studies, this study has the following advantages. First, this study actively explored the potential cause of PCA and investigated the relationship between SUA levels and PCA. Second, the data used in this study were obtained from the NHANES database. The data come from the real world and have the characteristics of standardization and large sample sizes. In addition, this study adjusted for different mixed factors, and a multi-regression analysis model was established for the data analysis to increase the accuracy of the results.

### 4.3 Limitations of this study

There are several limitations to this study. First, this study used a cross-sectional survey design. Therefore, causality cannot be inferred, and further prospective research should be conducted based on the findings of this study. Second, this study data came from the U.S. database, and whether the conclusions can be applied to other countries and regions requires additional research. Moreover, PCa data in this study were collected via self-reports rather than laboratory examinations; thus, there was some recall bias. Finally, although we adjusted for several potential influencing factors, some factors that were not considered may still impact the results.

## 5 Conclusion

PCa is one of the most common cancers worldwide and accounts for many cancer-related deaths. This study investigated the possible protective factors of PCa. The results of this study reveal one possibility: SUA can serve as an oxidant and has a certain potential protective effect on PCa, especially in some populations. However, additional basic research and prospective studies are needed to verify this conclusion.

## Data Availability

The original contributions presented in the study are included in the article/supplementary material. Further inquiries can be directed to the corresponding author.
